# Engineering the *Campylobacter jejuni* N-glycan to create an effective chicken vaccine

**DOI:** 10.1038/srep26511

**Published:** 2016-05-25

**Authors:** Harald Nothaft, Brandi Davis, Yee Ying Lock, Maria Elisa Perez-Munoz, Evgeny Vinogradov, Jens Walter, Colin Coros, Christine M. Szymanski

**Affiliations:** 1 Department of Biological Sciences, University of Alberta, Edmonton, Canada; 2 Alberta Glycomics Centre, University of Alberta, Edmonton, Canada; 3 Delta Genomics, Edmonton, Canada; 4 Department of Agricultural, Food & Nutritional Science, University of Alberta, Edmonton, Canada; 5 Human Health Therapeutics, National Research Council, Ottawa, Canada

## Abstract

*Campylobacter jejuni* is a predominant cause of human gastroenteritis worldwide. Source-attribution studies indicate that chickens are the main reservoir for infection, thus elimination of *C. jejuni* from poultry would significantly reduce the burden of human disease. We constructed glycoconjugate vaccines combining the conserved *C. jejuni* N-glycan with a protein carrier, GlycoTag, or fused to the *Escherichia coli* lipopolysaccharide-core. Vaccination of chickens with the protein-based or *E. coli*-displayed glycoconjugate showed up to 10-log reduction in *C. jejuni* colonization and induced N-glycan-specific IgY responses. Moreover, the live *E. coli* vaccine was cleared prior to *C. jejuni* challenge and no selection for resistant campylobacter variants was observed. Analyses of the chicken gut communities revealed that the live vaccine did not alter the composition or complexity of the microbiome, thus representing an effective and low-cost strategy to reduce *C. jejuni* in chickens and its subsequent entry into the food chain.

*Campylobacter* infections (primarily *C. jejuni* or *C. coli*) are among the most prevalent cause of human gastroenteritis worldwide^1^,^2^. Since *C. jejuni* is a common member of the chicken intestinal microbiome, poultry are major sources for human infection that results in the development of watery diarrhea, hemorrhagic colitis and in some cases reactive arthritis, Reiter’s syndrome, irritable bowel syndrome, and Guillain-Barré syndrome^3^,^4^. Thus, reducing *C. jejuni* at the source would significantly decrease the risk of human exposure and have a tremendous impact on food safety and public health.

Key prerequisites for antigens to be considered as vaccine candidates are immunogenicity and surface exposure. Attenuated campylobacter whole cell vaccines and nanoparticle encapsulated crude outer membrane protein lysates have been tested, but demonstrated limited protection^5^,^6^. More rational approaches included the use of specific protein antigens either purified, DNA-based or delivered by attenuated Salmonella strains. These include the flagellin subunit FlaA^7^,^8^, the outer membrane protein MOMP^9^, the adhesin Peb1^10^, the multidrug efflux pump component CmeC^11^, the ferric enterobactin receptor CfrA, the lipoproteins CjaA and CjaC (mediating amino acid transport)^12^, among others^13^,^14^,^15^,^16^,^17^,^18^,^19^. Although target-specific antibody responses were induced in most cases, the response provided either limited protection (FlaA-LTB^20^; rCmeC^21^; CjaD^22^ Dps^23^), was targeted against conformationally variable epitopes (MOMP)^24^,^25^, was not cross-protective (FlaA)^26^,^27^ or the results were highly variable (CjaA or CjaA-TetC)^22^,^28^,^29^,^30^,^31^ dependent on the model system or the route of administration. More recently egg yolk produced α-CadF, α-MOMP, and α-CmeC IgYs were suggested to be potentially useful as passive immunotherapeutics^32^, but their application did not result in a reduction of campylobacter colonization in chickens^33^.

Carbohydrates represent another class of biomolecules that have been successfully used for the generation of human glycoconjugate vaccines, but are currently not commercially available for animals^34^. *C. jejuni* is rich in surface carbohydrates including O- and N-linked glycoproteins^35^,^36^, capsular polysaccharides (CPS), and lipooligosaccharides (LOS); and studies using campylobacter CPS structures as antigens are showing promise in vaccine trials for human use^37^,^38^,^39^. However, since 47 different CPS serotypes have been identified for *C. jejuni* so far, the number of CPS types needed to achieve broad coverage against the most prevalent strains of *C. jejuni* needs to be determined and monitored for shifting populations^37^. Similarly, the variability in LOS and O-glycan structures limit the use of those carbohydrates as potential antigens. We were therefore interested in evaluating the use of the *C. jejuni* N-glycan as a vaccine candidate in chickens. The *C. jejuni* N-glycan is a heptasaccharide (GalNAc-α1,4-GalNAc-α1,4-[Glc-β-1,3]GalNAc-α1,4-GalNAc-α1,4-GalNAc-α1,3-diNAcBac; diNAcBac is 2,4-diacetamido-2,4,6-trideoxy-D-glucopyranose, GalNAc is N-acetylgalactosamine and Glc is glucose)^40^,^41^ that is common to all *C. jejuni* and *C. coli* isolates tested^35^,^36^. The N-glycan is constitutively expressed, added to multiple periplasmic and membrane proteins, protects the bacteria against proteolytic attack, is immunogenic in rabbits and humans, plays a role in innate and adaptive immunity, and is required for the colonization of mice and chickens, adherence and invasion of human epithelial cells and natural competence^35^,^42^,^43^,^44^. Moreover, the *C. jejuni* protein glycosylation (*pgl*) genes are transferable into heterologous hosts like *E. coli*^40^, to produce glycoproteins for biotechnological applications^44^.

Here we present two vaccine strategies. For the first approach, we created a glycoprotein that is comprised of a natural occurring *C. jejuni* peptide (GlycoTag, GT) that contains 9 perfect repeats of the bacterial N-glycosylation sequon (D/E-X1-N-X2-S/T, where X1 and X2 can be any amino acid but proline^45^) and is readily modified with up to 9 *C. jejuni* N-glycans when GlyoTag is fused to ToxC. In the second approach, a whole cell surface display system was used to fuse the N-glycan structure to the outer core of the *E. coli* lipopolysaccharide (LPS), replacing the natural O-antigen. Birds vaccinated with the GlycoTag-based or *E. coli* cell-surface exposed N-glycans showed N-glycan-specific immune responses and significant reductions in *C. jejuni* colonization levels after campylobacter challenge. The *E. coli* live vaccine was self-limiting and did not affect the composition of the chicken gut community thus providing an inexpensive and effective vaccination strategy to reduce *C. jejuni* in poultry.

## Results

### Expression of the protein-based *C. jejuni* N-glycan vaccine

**Figure 1:**
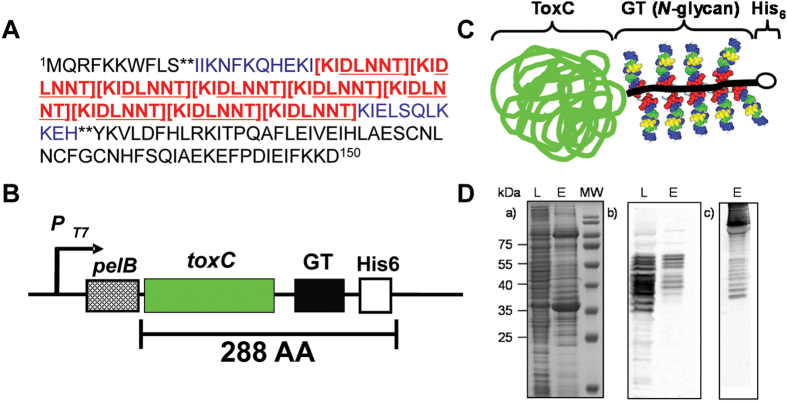
The protein-based *C. jejuni* N-glycan vaccine. **(A)** The first 150 amino acids of the *C. jejuni* Cj1433c polypeptide are shown. The sequence containing the GlycoTag peptide (nine repeats of [KIDLNNT]) is located between the double asterisks (**). The bacterial N-glycosylation sequon (DLNNT) is underlined. **(B)** Schematic diagram of the ToxC-GlycoTag-His_6_ expression construct: pT7, IPTG inducible promoter; *pelB*, pET22b-derived pectate lyase B leader sequence; *toxC*, truncated, non-toxic variant of the *C. diphtheria* toxin; His_6_, hexa-histidine-tag. **(C)** The anticipated N-linked glycosylated fusion protein is shown. (**D)** Expression and purification of glycosylated ToxC-GlycoTag-His_6_ in the presence of pACYC184 (*pgl*) in *E. coli* BL21 (DE3). (a) A 12.5% SDS-PAGE of whole cell lysates of BL21/ToxC-GlycoTag-His_6_ (L) and combined elution fractions after IMAC (E). Western blots with R1-4 antiserum of (a) combined elution fractions after IMAC, after (b) anion exchange chromatography and (c) after size exclusion chromatography. Molecular weight markers (in kDa) are indicated on the left.

To create an effective protein glycoconjugate for the one-pot synthesis of N-glycoproteins *in vivo*, we identified a novel campylobacter-derived N-glycan acceptor peptide, GlycoTag, located in the N-terminus of Cj1433. GlycoTag contains 9 perfect repeats of the amino acid sequence KIDLNNT including the bacterial N-glycosylation acceptor sequence DLNNT for the attachment of multiple *C. jejuni* N-glycans when expressed in *E. coli* (Fig. 1A). We genetically fused the GlycoTag to the C-terminus of a truncated and inactive variant of the *C. diphtheria* toxin, ToxC and further inserted a hexa-his coding sequence to the C-terminus and a *pelB* secretion signal to the N-terminus of the construct (Fig. 1B,C). The GlycoTag fusion was expressed in *E. coli* in the presence of the *C. jejuni* protein glycosylation (*pgl*) operon and purified to homogeneity (Fig. 1D, panels a and b). Western blot analysis confirmed modification of the GlycoTag fusion protein with up to 9 *C. jejuni* N-glycans (Fig. 1D, panel b and c). The carbohydrate content, based on the phenol-sulfuric acid assay, was calculated to be on average 3-4 N-glycans per molecule of protein that corresponded to approximately 150 μg N-glycan per mg of ToxC-GT-His_6_.

### Expression and validation of the whole cell *E. coli*-*C. jejuni* N-glycan vaccine

**Figure 2:**
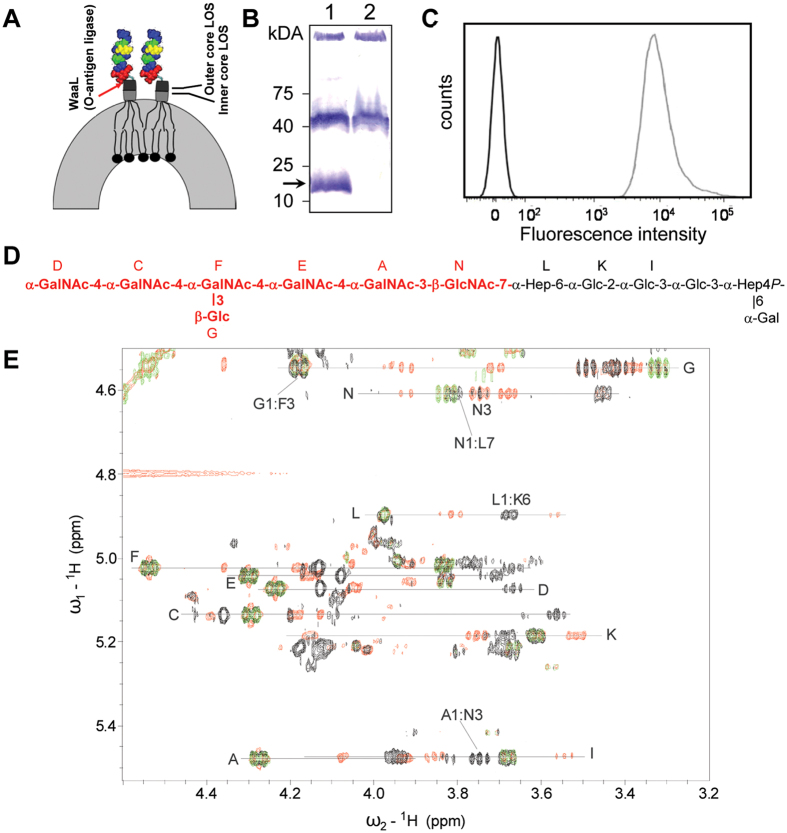
The *E. coli* cell surface display-based *C. jejuni* N-glycan vaccine. **(A)** Cartoon depicting the O-antigen ligase (WaaL)-dependent addition of the N-glycan structure to the LPS core of *E. coli*. **(B)** Western blot of proteinase K digested whole cell lysates of *E. coli wzy*::*kan* (pACYC184 (*pgl*_mut_)), lane 1 and *E. coli wzy*::*kan* (pACYC184), lane 2 probed with R1-4 are shown. The formation of the LPS-core-N-glycan molecule is indicated by an arrow. Molecular weight markers (in kDa) are indicated on the left. **(C)** FACS analysis of 2 × 10*4 cells of *E. coli wzy*::*kan* (pACYC184 (*pgl*_mut_)) in light grey and *E. coli wzy*::*kan* (pACYC184) in dark grey. Fluorescent intensity is shown on the x-axis, cell counts (arbitrary units) are shown on the y-axis. **(D)** The sequence of the LPS core N-glycan fusion product is shown. N-glycan-derived monosaccharide residues are shown in red. For the LPS-core part, only carbohydrate residues that could be assigned by NMR are shown. Capital letters refer to residues as outlined in Supplementary Table 2. **(E)** NMR spectrum of the purified LPS core-*C. jejuni*-N-glycan compound. Correlations from anomeric protons (as indicated) are shown. Green, COSY; red, TOCSY; black, ROESY.

To present the *C. jejuni* N-glycan on the *E. coli* cell surface, we fused the heptasaccharide to the outer LPS-core in an *E. coli* K12 O-antigen polymerase (*wzy*::*kan*) mutant background to avoid potential polymerization of the N-glycan structure (Fig. 2A). *E. coli* K12 does not produce endogenous O-antigen (O16) due to a naturally occurring mutation in the *wbbL* (rhamnosyltransferase) gene^46^. Western blotting with *C. jejuni* N-glycan-specific antiserum (R1-4) confirmed the formation of the LPS core-N-glycan fusion through the production of an immunoreactive signal present in proteinase K-treated *E. coli wzy*::*kan* pACYC184 (*pgl*_mut_) cells that migrated around 15 kDa (Fig. 2B, lane 1). This signal was absent in proteinase K-treated cells of the empty vector control, *E. coli wzy*::*kan* pACYC184 (Fig. 2B, lane 2). FACS analysis of 2 × 10^4^ formalin fixed cells probed with R1-4 and fluorescently labeled (Alexa546) secondary antibody showed a significant increase in fluorescence for *E. coli wzy*::*kan* (pACYC184 (*pgl*_mut_) cells compared to *E. coli wzy*::*kan* (pACYC184) (Fig. 2C). The peak appearance and geometry implies the presence of a comparable amount of the *C. jejuni* N-glycan on each *E. coli* cell.

The purified hybrid molecule was further analyzed by NMR confirming that a single unit of the *C. jejuni* N-glycan was fused to the *E. coli* LPS-core. Assignments for the N-glycan and the *E. coli* LPS-core part were in good agreement with published data^35^,^47^ (Fig. 2D,E, Supplementary Table 2): all 1–4- linkages of the *C. jejuni* N-glycan components gave transglycosidic Nuclear Overhauser effects of 1:4 and 1:6, all signals of the outer LPS-core l-*glycero*-d-*manno*-heptose (Hep, L in Table S2) were found by the analysis of the main heap of correlations and the N-glycan was linked to the O-7 of Hep (Fig. 2D). However, instead of diNAcBac that constitutes the native *C. jejuni* N-glycan reducing end sugar, GlcNAc was found at this position which has previously been observed when expressing the *C. jejuni* N-glycan structure in *E. coli*^48^.

### N-glycan based vaccines reduce *C. jejuni* colonization in chickens

**Figure 3:**
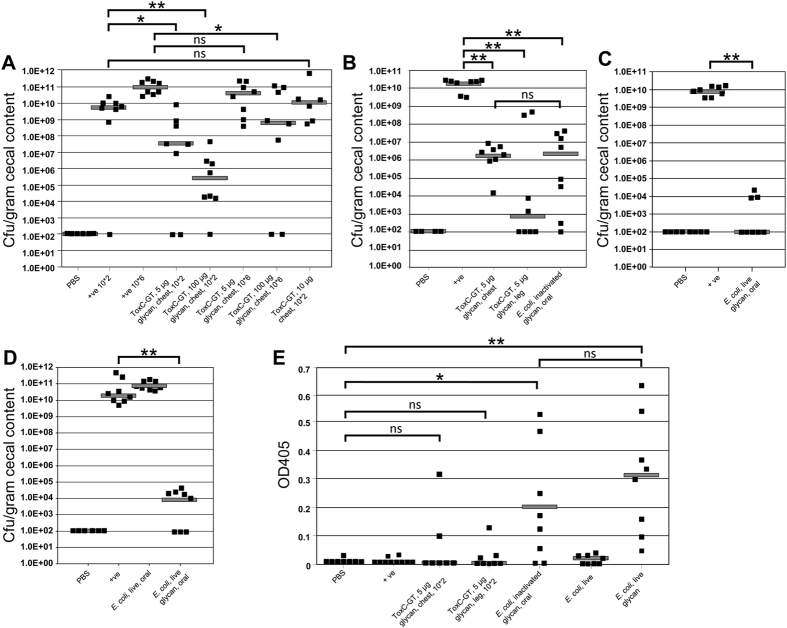
Chicken vaccinations and N-glycan-specific immune responses. **(A–D)** Colonization levels of *C. jejuni* 81–176 are shown. Each data point represents a single bird, grey bars represent the median level of *C. jejuni* colonization for each group expressed as CFU per gram of cecal material (detection limit, 1 × 10*2 CFU). Birds in the negative control groups (PBS) were non-vaccinated, non-challenged; birds in the positive control groups (+ve) were non-vaccinated but challenged with *C. jejuni* 81–176. **(A)** Treatment groups included birds that were vaccinated by injecting either 100 μg or 5 μg of glycosylated ToxC-GT-His_6_ protein or non-glycosylated ToxC-GT-His_6_ protein (control) into the breast on days 7 and 21 before challenge with 10*2 or 10*6 CFU (as indicated) of *C. jejuni* 81–176 on day 28. (**B**) Treatment groups included birds that were vaccinated by injecting 5 μg of glycosylated ToxC-GT-His_6_ protein in the breast or in the leg on day 7 and 21 and in parallel, birds that were orally gavaged with 10*8 inactivated *E. coli* cells expressing the *C. jejuni* N-glycan on their surface before challenge with 10*2 CFU of *C. jejuni* 81–176 on day 28. **(C)**. Birds in the treatment group were orally gavaged on day 7 and 21 with 10*8 live *E. coli* cells expressing the *C. jejuni* N-glycan on their surface before challenge with 10*2 CFU of *C. jejuni* 81–176 on day 28. **(D)** Birds in the treatment groups were orally gavaged on day 7 and 21 with either 10*8 live *E. coli* cells not expressing the N-glycan (control group) or 10*8 live *E. coli* cells expressing the *C. jejuni* N-glycan on their surface before challenge with 10*2 CFU of *C. jejuni* 81–176 on day 28. **(E)** N-glycan-specific antibody responses. ELISA results using a 1:10 dilution of chicken sera from bleeds taken prior to *C. jejuni* challenge (day 28). Each point represents the antibody response measured at OD_405_ for each individual chicken. Grey bars represent the median for each group. **(A–E)** Statistical differences between groups are indicated: ns, no statistically significant difference (*p*-value < 0.05); * and **indicate statistically significant differences with *p*-values > 0.05 and >0.005, respectively.

In a 35-day SPF Leghorn chicken challenge model, we tested the efficacy of each vaccine composition as well as the best dosage and route of administration. The glycoprotein vaccines were injected into the breast or in the leg while whole cell vaccines (live or inactivated) were orally gavaged. First, we determined the *C. jejuni* challenge and the protein glycoconjugate doses (Fig. 3A). Birds that were challenged with 1 × 10*2 and 1 × 10*6 *C. jejuni* cells on day 28 showed comparable colonization levels. Vaccination in the breast on days 7 and 21 with 5 μg or 100 μg of purified glycosylated ToxC-GT protein resulted in a statistically significant reduction in bacterial colonization (p-values < 0.05 and <0.005, respectively) after challenge with 1 × 10*2 *C. jejuni*. In the treatment groups that received a higher *C. jejuni* challenge dose (1 × 10*6), the bacterial load was significantly reduced in birds that received the higher vaccine dose (p-value > 0.05). Birds receiving the lower vaccine dose showed no statistically significant difference compared to the non-vaccinated group (p-value > 0.05) Similarly, vaccination with non-glycosylated ToxC-GT led to similar colonization levels when compared to the positive control group (Fig. 3A). No *C. jejuni* above the detection limit of 1 × 10*2 CFU/gram cecal content was observed in negative control birds. Next we tested if the injection site influences the efficacy of the protein vaccine (Fig. 3B). The colonization level after challenge with 1 × 10*2 *C. jejuni* dropped significantly from 2 × 10*10 in the positive control birds to 2 × 10*6 (p-value < 0.005) and 9 × 10*2 (p-value < 0.005) colony forming units (CFU) per gram cecal content in birds vaccinated in the chest or in the leg on days 7 and 21. Levels of *C. jejuni* in negative control birds were below the detection limit. In comparison, we also tested the efficacy of inactivated *E. coli* cells displaying the N-glycan that were administered by oral gavage. For this treatment, a statistically significant drop (p-value < 0.005) in the *C. jejuni* load after challenge to 3 × 10*6 CFU per gram cecal content was observed (Fig. 3B). Subsequently, we tested the live *E. coli* vaccine. In two independent studies, birds were orally vaccinated with the *E. coli* strain expressing the LPS core-N-glycan on its surface. *C. jejuni* colonization after challenge was significantly reduced (p-value < 0.005) when compared to colonization levels in unvaccinated birds (Fig. 3C,D). In contrast, no statistically significant difference (p-value > 0.05) in the colonization levels was observed in birds that received the isogenic *E. coli* strain not expressing the *C. jejuni* N-glycan structure on its surface (Fig. 3D).

We also determined the relative levels of *E. coli* in birds that received the live vaccine. No *E. coli* was detected prior to the first *E. coli* gavage (day 7). The levels of *E. coli* declined when monitored at 2, 5 and 9 days after the first vaccination (Supplementary Table 3 and Supplementary Methods). A more rapid reduction of *E. coli* was observed after the second gavage (day 21) with no detectable *E. coli* by day 28 for the vaccine strain and low levels of *E. coli* in birds that were given the control strain. No *E. coli* was detected on day 35 clearly showing that the vaccine strain is self-limiting. Interestingly, the live *E. coli* vaccine expressing the N-glycan appeared to be cleared faster when compared to the *E. coli* control strain.

### Vaccinated birds develop an N-glycan specific IgY response

N-glycan-specific immune responses were determined in sera taken at day 28 prior to *C. jejuni* challenge (Fig. 3E). The average immune response against each vaccine corresponded to the degree of protection against *C. jejuni* colonization; however, the highest individual titres did not correlate with birds showing the lowest levels of *C. jejuni* colonization. The highest titres with a statistically significant increase in the IgY levels when compared to negative control birds, were observed in birds vaccinated with the live *E. coli* strain expressing the N-glycan (p-value < 0.005) on its surface followed by birds that received the inactivated strain (p-value < 0.05). However, the increase in the median between these two groups was not statistically significant (p-value > 0.1). Although some birds that received the protein glycoconjugates also showed an increase in the N-glycan-specific IgY levels when compared to negative control birds, the comparison of the median did not result in an overall statistically significant increase (p-values > 0.05). No detectable N-glycan-specific antibody titres were present in non-vaccinated birds, in birds that received the *E. coli* strain not expressing the N-glycan, and in birds that received non-glycosylated ToxC-GT-His_6_ (not shown) indicating that the observed increase in IgY titres was due to the presence of the *C. jejuni* N-glycan on either the surface of *E. coli* or when N-linked to the ToxC-GT His_6_ protein.

### Vaccination of chickens does not select for *C. jejuni* resistant strains

Although some birds vaccinated with the live *E. coli* N-glycan expressing strain showed non-detectable levels of *C. jejuni* colonization, others still had low levels of colonization (Fig. 3C,D). Those isolated *C. jejuni* colonies were probed with the N-glycan-specific R1-4 antiserum (Supplementary Fig. 1). Every colony showed strong reactivity with the antiserum verifying that these isolates still express the N-glycan and that no selection for *C. jejuni* variants lacking the N-glycan structure took place (Supplementary Fig. 1). Whole cell lysates of the wild-type, spotted as a positive control resulted in a similar spot intensity when visually compared to the N-glycan positive colonies, whereas only background reactivity was observed when spotting whole cell lysates of the *C. jejuni* N-glycosylation mutant (*pglB*) indicating that the observed reactivity is indeed dependent on the presence of the N-glycan.

### Vaccination prevents *C. jejuni* colonization without changes in the resident bacterial community in the Leghorn chicken intestine

**Figure 4:**
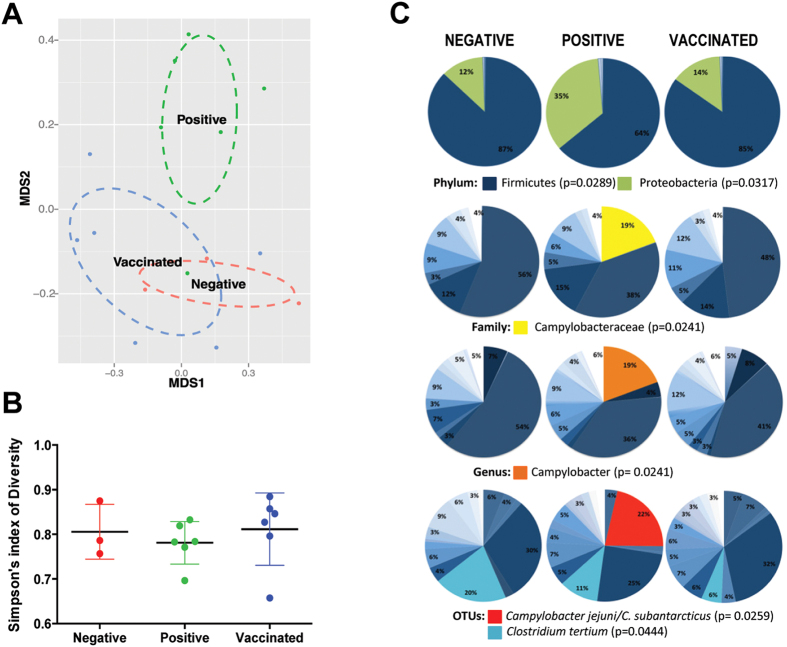
Vaccine decreases *Campylobacter* species to original levels and does not alter the global microbial community in the Leghorn gut. **(A)** NMDS ordination plot of cecal bacterial communities based on the Bray-Curtis distance metric. Colonization by *C. jejuni* (positive group) causes a shift in the global microbial composition of the Leghorn gut, while the bacterial community structure in vaccinated birds resembles that of birds from the negative control group. **(B)** Vaccine treatment causes no changes in alpha diversity as measured by the Simpson index of diversity. **(C)** Composition of the cecal bacterial community at phylum, family, genus, and species (OTU) level.

In birds from the positive control groups that were initially inoculated with 1 × 10^2^ CFU of *C. jejuni* and subsequently showed colonization levels up to 1 × 10*10 CFU/gram cecal content, the presence of *C. jejuni* caused a shift in the global structure of the resident bacterial communities, as shown in a non-metric multidimensional scaling (NMDS) ordinance plot based on Bray Curtis metrics (Fig. 4A) and PCoA on weighted UniFrac metrics (data not shown). Vaccination with the live *E. coli*-based vaccine reversed these changes, causing the gut microbiota of vaccinated birds to shift back to the composition observed in the negative control group that did not receive *C. jejuni*. Assessment of alpha diversity (within samples) showed no significant differences between these two treatment groups (Fig. 4B). Inoculation with *C. jejuni* led to major colonization by the species, leading to a significant increase in the relative abundance of microbes from the phylum Proteobacteria (increased from 12% to 35%), the family *Campylobacteracae* (increased from less than 0.001 to 19%), the genus *Campylobacter* (increased from less than 0.001% to 19%) and the species (OTU) *C. jejuni/C. subantarcticus* (increased from less than 0.001% to 22%) (Fig. 4C) demonstrating that *C. jejuni* establishes itself in the chicken gut without decreasing diversity or changing the resident community, supporting its non-pathogenic status. Vaccination led to a substantial reduction in colonization of *C. jejuni* (less than 0.001%, p < 0.05), supporting our culture-based findings. Few other significant changes in the microbiota were detected. An OTU related to *Clostridium tertium* decreased from 20% in the negative group, to 11% in the positive, and further reduced to 6% in the vaccinated group (p = 0.0444) (Fig. 4C).

## Discussion

Three major strategies for reducing *C. jejuni* infection in poultry have been identified^49^: (1) reduction of environmental exposure (e.g. biosecurity measures), (2) measurements to decrease *C. jejuni* in the chicken gut (e.g. vaccination), and (3) the use of antimicrobial alternatives (e.g. bacteriophage therapy or bacteriocin treatment). Except for biosecurity measures, these approaches are still under development. Active immunization of poultry would be an attractive alternative to administering antibiotics to decrease the abundance of *C. jejuni* in the native host and the resulting diarrheal disease in humans.

An effective vaccine against *C. jejuni* in poultry has to meet three main challenges: (1) the identification of cross-protective antigens, (2) the induction of a rapid and strong immune response, and (3) the development of novel adjuvants to further stimulate immunity against *C. jejuni*^50^. The *C. jejuni* N-glycan fulfills all of these requirements. It is surface exposed^35^,^42^, immunogenic in humans and rabbits^35^,^51^,^52^ and, as demonstrated in this study, induces a protective immune response in chickens. In addition, lipid A present in the live *E. coli* vaccine as well as the use of the toxoid, ToxC, in the ToxC-GT-*C. jejuni* N-glycan-His_6_ glycoconjugate act as natural adjuvants to stimulate the immune system. Since the N-glycan is the only glycoconjugate structure conserved among all *C. jejuni* isolates^35^,^36^,^53^, we would expect the N-glycan specific immune response to be cross-protective.

Both, our recombinant glycoprotein (GlycoTag) and whole cell delivery approaches result in a multivalent presentation of the N-glycan. Multivalent presentation of group B streptococcus carbohydrate epitopes was demonstrated to be significantly more efficient than currently available vaccines that have a lower carbohydrate to protein ratio^54^. Similarly, a vaccine with two to five CPS per CRM197 was sufficient to induce a protective immune response in mice and monkeys against challenge with *C. jejuni* 81–176^39^. Although we observed a reduction in *C. jejuni* colonization with the administration of higher doses of the glycoconjugate vaccine after challenge with 10*2 as well as 10*6 *C. jejuni* CFU, the lower challenge dose is probably more reflective of the natural conditions when *C. jejuni* is first introduced into the flock, e.g. through flies that enter the poultry houses^55^,^56^. Artificial fly feeding studies demonstrated that *C. jejuni* levels are not higher than 1 × 10*4 CFU^57^ and it has been shown that as low as 40 colony forming units of *C. jejuni* are sufficient to induce chicken colonization, however, the infectious dose varies between strains of *C. jejuni*^58^,^59^.

The presentation of the N-glycan on the *E. coli* cell surface is possible due to the interplay between the endogenous O-antigen LPS and the heterologous N-glycan biosynthesis pathways^60^,^61^,^62^ and their requirement for undecaprenylphosphate for sugar assembly. Interestingly, WecA, the initiating GlcNAc transferase involved in enterobacterial common antigen and O-antigen biosynthesis can substitute for *C. jejuni* PglC function however, preferring UDP-GlcNAc rather than UDP-diNAcBac as the initiating monosaccharide^61^. Although *pglC* (on pACYC184*pgl*_mut_) and the chromosomal copy of *wecA* are both present in the live vaccine strain and it has been shown that diNAcBac and GlcNAc containing *C. jejuni* N-glycan lipid-linked oligosaccharides (LLOs) were produced simultaneously when the *pgl* locus is expressed in *E. coli*^48^, only GlcNAc was found to be the linking sugar to the O-7 of the l-*glycero*-d-*manno*-heptose of the LPS-core. One explanation might be that although the *E. coli* K-12 WaaL O-antigen ligase has been reported to lack substrate specificity^63^, GlcNAc containing N-glycan LLOs are preferred over diNAcBac containing LLOs. One might argue that the absence of diNAcBac could negatively influence the protective immune response against the *C. jejuni* N-glycan, but we have previously demonstrated that the immune response against the N-glycan is targeted against the non-reducing end residues^35^,^64^,^65^,^66^.

The live *E. coli* vaccine strain confers better protection against *C. jejuni* compared to the inactivated *E. coli* likely due to longer antigen exposure times in the chicken and through the induction of a stronger mucosal immune response by a live carrier. The fact that live *E. coli* expressing the N-glycan are cleared faster after the second (booster) dose compared to *E. coli* alone might be a result of this N-glycan specific immune response induced by this strain, thus minimizing the risk of meat contamination with the vaccine strain. The live *E. coli* vaccine prevents *C. jejuni* colonization without altering the indigenous chicken gut microbiota, suggesting a specific immune response with no adverse effects on microbiome composition.

We demonstrated that the N-glycan was still present on all *C. jejuni* isolates after passage through vaccinated birds indicating there was no selection for N-glycan-negative *C. jejuni* variants. This is not surprising since the *pgl* genes are lacking homopolymeric tracts that are subject to high-frequency slip-strand mutation as shown for genes encoding *C. jejuni* O-linked glycans, LOS and CPS structures^37^,^67^,^68^,^69^,^70^,^71^. In the unlikely event that selection against the N-glycan would occur, these cells would not be able to survive in the chicken gut since the N-glycan itself is required for chicken colonization and protects the cell from proteolytic attack by chicken gut proteases^35^,^42^,^43^,^44^.

The use of the conserved N-glycan structure in vaccine compositions significantly reduces *C. jejuni* at the source. We show that treatment with protein-based glycoconjugates significantly reduces *C. jejuni* colonization after challenge with the organism independent of the injection time point or the application site. Oral vaccination with live *E. coli* cells expressing the N-glycan on their surface significantly reduced colonization. The overall levels of IgY antibodies were in agreement with the level of protection after challenge indicating that the response is protective.

The use of live *E. coli* as a self-limiting carrier for the N-glycan antigen is favorable over previously used Salmonella-based delivery systems that might prove difficult to introduce with respect to food standards in certain countries. In addition, the *E. coli* vaccine is easy to produce and to administer compared to the use of individual protein antigens that have to be purified large-scale and potentially administered by subcutaneous injections to reach their full potential. These facts will allow the testing of the vaccine in large scale applications using other popular breeds of chickens for the creation of a low cost vaccine for *C. jejuni* reduction.

## Methods

### Bacterial strains, plasmids and growth conditions

*C. jejuni* strain 81–176 was grown on Mueller Hinton (MH) agar (Difco) at 37 °C under microaerobic conditions (85% N_2_, 10% CO_2_, and 5% O_2_). *Escherichia coli* strains were grown on Luria Bertani (LB) or 2-times YT (2xYT) medium supplemented with ampicillin (Amp), kanamycin (Km), or chloramphenicol (Cm) at a final concentration of 100, 50 or 20 μg/ml where needed. Karmali supplement (if required) was added according to the instructions of the manufacturer (Oxoid). Bacterial strains and plasmids are summarized in Supplementary Table 1.

### Bioinformatic analyses of the *C. jejuni* proteome

FASTA protein sequences from *C. jejuni* species available from the EMBL server (http://www.ebi.ac.uk/) were used to perform an amino acid motif search using the protein pattern find software Sequence Manipulation Suite: Protein Pattern Find (http://bioinformatics.org/sms2/protein_pattern.html, with (d|e).n.(s|t) as the search criteria that matches the requirement for the bacterial N-linked glycosylation site D/E-X1-N-X2-S/T. Since positions X1 and X2 do not tolerate a proline^45^, obtained sequences were manually investigated for the occurrence of this amino acid and excluded if present. The annotations/putative functions of the remaining proteins were subsequently (manually) searched for the keywords “periplasmic”, “membrane” and “secreted proteins” and proteins were sorted according to the frequency of glycosylation sites present.

### Cloning, expression and validation of the glycosylated GlycoTag fusion protein

The gene encoding an enzymatically inactive and nontoxic form of the diphtheria toxin (toxoid, *toxC*) from *Corynebacterium diphtheriae* was amplified from plasmid pPDT1^72^ with oligonucleotides CS-378 (5′- ATATATATCCATGGCTGCTGATGATGTTGTTGATTC-3′) and CS-379 (5′- ATATACTCGAGTCGCCTGACACGATTTCCTGCACAGG3′) to introduce NcoI and XhoI sites, respectively. The obtained NcoI-XhoI digested PCR product was inserted into plasmid pET22b cut with the same enzymes translationally fusing the gene to the plasmid-derived *pelB* secretion sequence for the transport of the product into the periplasmic space. A 271 bp DNA fragment including the 9 N-glycosylation sequon repeat (GlycoTag, GT) was amplified from chromosomal DNA of *C. jejuni* 11168 with oligonucleotides CS-334 (5′ AAACTCGAGTTCATAAAAAATTTCAAGC3′) and CS-335 (5′ ATATCTCGAGCTCTTTTTTTAATTGCG3′) inserting XhoI sites in the 5′ and 3′ prime ends. To fuse the GlycoTag sequence to the C-terminus of ToxC, the XhoI digested PCR product was inserted into plasmid pET22b*toxC*, linearized with XhoI, and dephosphorylated with shrimp alkaline phosphatase (SAP). The orientation of the GT sequence was confirmed by sequencing. This resulting construct expresses the *pelB*-*toxC*-GT fusion including a C-terminal pET22b derived Hexa-Histidine (His_6_) tag. Protein expression was performed in *E. coli* BL21(DE3) in the presence of plasmid pACYC184 (*pgl*)^40^. An overnight culture was used to inoculate 1 litre of 2xYT broth to an OD_600_ of 0.1. Cells were grown at 37 °C until an OD_600_ of 0.6 was reached. Cells were cooled on ice for 30 min, protein expression was induced by addition of IPTG to a final concentration of 0.5 mM, and cells were grown for an additional 18 hrs at 30 °C. Cells were cooled on ice, harvested by centrifugation (15 min 4,200 × g, 4 °C), and resuspended in PBS supplemented with an EDTA free protease inhibitor cocktail according to the instructions of the manufacturer (Roche). Cells were disrupted in a cell disrupter (Constant Systems, Ltd), the resulting suspension was centrifuged for 30 min at 13,000 × g, 4 °C, and the resulting supernatant was loaded onto a 1 ml Ni-NTA column using the AEKTA purification system (GE Healthcare). After an initial wash step with 10 mM imidazole in PBS, an imidazole gradient was applied from 10–250 mM over 50 column volumes. Elution fractions that contained the ToxC-GT-His_6_ protein were analyzed by 12.5% SDS-PAGE, combined and the glycosylation status of ToxC-GT-His_6_ was verified by Western blotting as described previously^35^. The ToxC-GT-His_6_ proteins were dialyzed against 25 mM potassium phosphate buffer, 10 mM NaCl, pH 7.2, and further purified by anion exchange chromatography on a 2.5 ml MonoQ column (GE Healthcare) with a 100 ml linear gradient of NaCl (10–500 mM) in 25 mM potassium phosphate, pH 7.2. Fractions containing ToxC-GT-His_6_ were desalted by size exclusion chromatography on a Sepahadex 75 column using PBS as the mobile phase. Fractions that contained the target protein as determined by 10% SDS-PAGE and Western blotting with R1-4 antisera were combined. The protein concentration was determined using the BioRad DC protein assay kit according to the instructions of the manufacturer and adjusted to 0.2 mg/ml. The N-glycan amount per protein was calculated after determination of the carbohydrate content of ToxC-GT-His_6_ using the colorimetric phenol-sulfuric assay^73^ and known concentrations of purified fOS (free oligosaccharides)^74^ as a standard. If necessary, centrifugal filters (Amicon, 10 kDa cut-off) were used to concentrate the proteins. Proteins were stored at 4 °C until further use.

### Preparation of *E. coli* cells for downstream processing

*E. coli* K12 *wzy*::*kan* (KEIO collection^75^) was transformed with plasmids pACYC184 (*pgl*_mut_) and pACYC184. Whole cells for vaccination and verification of antigen expression were prepared as follows: overnight cultures were used to inoculate fresh 2xYT broth to an OD_600_ of 0.1. Cells were grown at 37 °C without antibiotics until an OD_600_ of at least 1.0 was reached. Cells were cooled on ice (10–15 min), harvested by centrifugation (5 min 4,200 × g, 4 °C), washed twice in ice-cold PBS and adjusted to an OD_600_ of 1.0 using the same buffer.

Cell counts were determined as follows: aliquots of a 10-fold dilution series (prepared in PBS) of cells adjusted to an OD_600_ of 1.0 were plated on 2xYT plates containing Km and Cm. Colony forming units (CFUs) were in a range of 0.9 to 1.1 × 10*8 bacteria per 1 ml of OD_600_ = 1.0 cells. To adjust the vaccination dose, cells were resuspended in 1/3 of the original OD_600_ = 1.0 volume resulting in 3 × 10*8 cells per ml (or 1 × 10*8 cells in 300 μl = one dose). Alternatively cells were cross-linked/fixed using 1% formaldehyde in PBS for 1 h at 4 °C as previously described^76^, centrifuged (5 min 4,200 × g, 4 °C), and washed 4-times with ice-cold PBS. Cross-linked cells were resuspended in PBS that corresponded to 1/3 of the original volume of the OD_600_ = 1.0 cell suspension to obtain 3 × 10*8 cells/ml (that equals 1 × 10*8 cells in 300 μl that were needed for one dose of the inactivated whole cell-based vaccine).

### Preparation of protein free cell extracts

Glycolipid extracts were prepared as previously described^77^. Briefly, *E. coli* cells of a culture equivalent to an OD_600_ of 1.0 (prepared as described above) were centrifuged, resuspended in 100 μl of 1 × Laemmli sample buffer, and heated to 95 °C for 10 min. Proteinase K (Fermentas) was added to a final concentration of 200 μg/ml and the sample was incubated at 60 °C for 1 h. Glycolipid species from the proteinase K-digested whole cell lysates were separated by 12.5% SDS-PAGE, transferred to PVDF membranes and analyzed by Western blotting as previously described^35^.

### Cross absorption of R1 antiserum

*C. jejuni* N-glycan-specific antiserum (R1^35^) was cross absorbed using whole cells of *E. coli wzy*::*kan* (pACYC184) as follows: the pellet of 1 ml of OD_600_ = 1.0 culture was blocked with 1 ml PBS and 5% skim milk for 20 min. Cells were spun for 5 min at 4,200 × g, resuspended and incubated with 1 ml of R1 serum for 30 min on ice with occasional inversion of the tube. Cells were spun out of the mixture and the supernatant was used to repeat the procedure 4X. The resulting serum (R1-4) that was depleted of *E. coli*-specific antibodies was used for downstream analyses.

### Western blotting

Western blots were performed as previously described^35^. *C. jejuni*-N-glycan-specific antiserum, R1, or cross-absorbed R1 (R1-4) was used at a 1:7,500 dilution, anti-His antiserum (Santa Cruz Biotech) was used at a 1:1,000 dilution, and AP-conjugated rabbit and mouse antisera (Santa Cruz Biotech) were used at 1:2,000 dilutions. Immunoreactive bands were visualized directly on the PVDF membrane using the NBT-BCIP detection reagents (Promega) according to the instructions of the manufacturer.

### Colony lifts

Cecal content dilutions were plated on MH agar supplemented with the Karmali supplement and Trimethoprim. Colony lifts were performed as previously described^78^. Immunodetection was done as for Western blotting (described above).

### Fluorescence activated cell sorting (FACS)

*E. coli* cells were adjusted to OD_600_ of 1.0 and 1 ml was pelleted by centrifugation and resuspended in 1 ml blocking solution (PBS, 5% skim milk). Cells were probed with R1-4 and Alexa Flour-546 conjugated anti-rabbit antiserum, and analyzed by FACS (on a LSR-Fortessa Flow Cytometer). FACS data were processed with the FACS Diva software. DAPI counter-staining was used to identify and gate for intact cells.

### NMR

Glycolipids were prepared from cell pellets obtained from eight litres of OD_600_ = 1.0 *E. coli wzy*::*kan* (pACYC184*pgl*_mut_) cells grown as described above. LPS was extracted by phenol-water, dialyzed, treated with acetic acid (AcOH) to precipitate nucleic acids, dialyzed, dried, hydrolyzed with 2% AcOH and separated on Biogel P6. Fractions were analyzed by NMR. Fractions that contained *C. jejuni* N-glycan signals were combined and separated on an anion-exchange Hitrap column using an NaCl gradient. Fractions were again analyzed by NMR. *C. jejuni* N-glycan LPS-core components eluted as a broad peak after the enterobacterial common antigen peak (data not shown). Fractions containing *C. jejuni* N-glycan signals were desalted by Sephadex G-15 chromatography. Connections were confirmed by NOE and HMBC.

### Chicken challenge studies

Animal studies were carried out in accordance with the protocol approved by the Animal Care and Use Committee at the University of Alberta using a 35 day challenge protocol. In general each group contained up to 8 birds (SPF Leghorns, Poultry Research Facility, University of Alberta) that were randomly tested for the presence of *C. jejuni* on the day of hatch (day 1) by plating cloacal swabs onto selective Karmali agar. In all cases no *C. jejuni* colonies were observed on plates after 48 hr of incubation under microaerobic conditions at 37 °C.

#### Chicken vaccination

To test the efficacy of the protein glycoconjugates, birds received 300 μl of protein antigen prepared 1:1 in Freund’s complete adjuvant for the 1^st^ vaccination (day 7) and the same amount but with Freund’s incomplete adjuvant for the 2^nd^ vaccination (day 21). Antigens were injected at two sites in the chest with 150 μl of vaccine formulation per site or in the leg with 150 μl of vaccine formulation in each leg. Control groups received PBS in Freund’s complete/incomplete instead of protein. Vaccination with whole cells of *E. coli* was done by orally gavaging 300 μl of PBS containing 1 × 10*8 live or inactivated (cross-linked, as described above) *E. coli* cells on days 7 and 21. Control groups were gavaged with 300 μl of PBS only. In the case of the *E. coli* whole cell live vaccine, cloacal swabs taken at various time points were plated onto LB Km-Cm. Relative CFUs for each bird were determined by colony counts after 18 hr of incubation at 37 °C.

#### Campylobacter challenge

Birds were orally gavaged (challenged) on day 28 with either PBS (negative control) or with 300 μl PBS containing 10*2 or 10*6 *C. jejuni* strain 81–176 cells. To prepare the challenge, *C. jejuni* 81–176 was grown for 18 h on MH agar and harvested with cold MH broth. Cells were washed twice with cold PBS and adjusted to an OD_600_ of 1.0 (OD_600_ of 1.0 equals 1.5 × 10*9 cell/ml). Serial dilutions in PBS were performed dependent on the final amount of cells that were administered. For example: 3 × 10*2 cells/ml (=1 × 10*2 cells per 300 μl =1 dose). Cells were maintained on ice until used. Birds were maintained for 7 days after challenge and then euthanized according to the approved guidelines of the Canadian Council for Animal Care. Ceca were collected, the contents were removed and weighed and adjusted to 1 mg cecal content per 1 ml with sterile PBS. Aliquots of 10-fold serial dilutions (in PBS) of the cecal contents were plated on selective Karmali agar. CFU were determined after incubation of the plates for 48 hr under microaerobic conditions.

#### Serum preparation

Blood samples were collected on day 7 (50 μl pre-bleed) and day 28 (100 μl vaccine response prior to challenge). Fresh blood samples were kept at room temperature for at least 18 hr or at 37 °C for at least 6–8 hr, until a firm blood clot was formed. Samples were centrifuged (5 min, 13,000 × g, 4 °C) and the supernatant (serum) was transferred to a fresh tube. After addition of glycerol to a final concentration of 10%, the sera were stored at −20 °C until further use.

### ELISA testing for N-glycan-specific antibodies

We developed a 96-well plate ELISA assay to quantify the N-glycan-specific IgY responses in vaccinated birds. fOS from *C. jejuni* (Cj) was prepared as described^74^ and coupled to BSA by reductive amination as previously described^35^. Formation of the BSA-Cj-N-glycan conjugate was confirmed by Western blotting using R1-4 antiserum. After adjusting the concentration to 1 mg/ml using PBS, the glycoconjugate was stored at 4 °C until further use. We first tested the BSA-Cj-N-glycan conjugate binding capacity by coupling increasing amounts of the antigen and probed with R1-4 antiserum. A linear increase in signal intensity was observed over a range of 5 to 500 ng of the capture antigen (data not shown). No increase in signal intensity was observed when higher concentrations of the BSA-Cj-N-glycan conjugate were added to each well, therefore 500 ng of BSA-Cj-N-glycan conjugate per well were used for further analyses.

Then, 96-well Maxisorb plates (Thermo Fisher) were coated with 500 ng of BSA-Cj-N-glycan conjugate overnight (18 hr) at 4 °C. After removal of unbound antigen, the plate was blocked for 1 hr at RT with 5% skim milk in 100 μl PBS-T with shaking. After discarding the blocking solution, 100 μl of the antibody solution was added and incubated for 1 hr as described above. Antibody solutions were R1-4 antiserum diluted 1:3000 in PBS-T with 1% skim milk, or chicken serum (prepared from day 28 bleeds from the 2^nd^ vaccination experiment, Fig. 3B), diluted 1:10 in PBS-T with 1% skim milk. Plates were incubated for 1 hr at RT as described and each well was washed 3 times for 5 min with 100 μl of PBS-T. After addition of 100 μl of the secondary antibody solution (either anti-rabbit-AP (1:500), for the R1-4 control or anti-chicken IgY (1:500) for the experimental samples and incubated for 1 hr at RT), the secondary antibody solutions were discarded and the wells were washed 4 × 5 min with 100 μl of PBS-T. After the last washing step, the remaining washing solution was completely removed from each well and the plates were developed using pNPP as a substrate following the instructions of the manufacturer (Thermo Fisher). Immunoreactivity in each serum was determined after scanning the plate at OD_405_ in a plate reader.

### Chicken microbiome studies

Chicken microbiome studies were performed to analyze and compare the composition of the bacterial community in negative control (non-vaccinated, not challenged) and positive control (non-vaccinated, challenged) birds in comparison with birds that were challenged with *C. jejuni* after they received the *E. coli* live vaccine strain that expresses the N-glycan on its surface.

#### DNA isolation

First, 250–300 mg of cecal contents were placed in a 2 ml tube and washed with cold 1xSTE buffer (100 mM NaCl, 1 mM EDTA, 10 mM Tris/HCl pH 8.0). The sample was spun at low speed (1,000 rpm) to remove large pieces of unwanted debris. The supernatant was placed in a new 2 ml tube and spun at a higher speed 14,500 rpm on the mini-spin) to pellet the bacteria in the sample. The supernatant was removed; the pellet was resuspended by vortexing and was washed twice with 1 ml of ice cold 1xSTE buffer. After removing the supernatant, 180 μl of Qiagen ATL was added with 20 ml of Roche PK and digested overnight at 56 °C on a rotisserie. The DNA was extracted on the Biosprint using the Biosprint_96_DNA tissue and blood kit© according to the QIAGEN protocol. The DNA samples were quantified using the Promega QuantiFlour®dsDNA System kit.

#### Library preparation and quantification

Extracted DNA from chicken cecum samples was initially amplified using the universal primers 926F (5′-AAACTYAAAKGAATWGRCGG-3′) and 1392R (5′-ACGGGCGGTGWGTRC-3′) that targets the V6 to V8 region of the 16S ribosomal RNA gene with PCR conditions as detailed in the 16S Metagenomic Sequencing Library Preparation (Illumina®, San Diego, CA). A bioanalyzer trace of amplified products was obtained using the DNA 1000 chip on Agilent 2100 Bioanalyzer (Agilent Technologies, Santa Clara, CA). Amplicons with a single product at approximately 500 bp were determined to be suitable for further library preparation. Subsequently PCR cleanup was carried out using the Agentcourt Ampure XP beads (Beckman Coulter, Mississauga, ON). Nextera XT Dual indexing barcodes adapters (Illumina®) were attached to the bead-cleaned amplicons by a second PCR as detailed in the 16S Metagenomic Sequencing Library Preparation (Illumina®). Barcoded amplicons were cleaned up by an additional step of Agentcourt Ampure XP bead clean up (Beckman Coulter). ABI Veriti 96 well Thermal Cycler (Life Technologies, Burlington, ON) was used to run all the PCR reactions. Library quality was assessed by running the DNA1000 chip on the Agilent 2100 Bioanalyzer (Agilent Technologies). Library sizes ranged from 630 to 670 bp. Qubit HS dsDNA Assay (Life Technologies) was used to quantify the libraries. Individual libraries with their respective barcodes were pooled in a 4 nM library pool. The 16S rRNA gene sequencing was carried out on the Illumina MiSeq sequencer with the MiSeq Reagent kit V3 (Illumina) generating 300 bp reads in both the forward and reverse directions.

#### Microbiome - data analysis

Illumina 16S rRNA sequence reads were processed and analyzed as previously described^79^ with minor modifications as follows. Reads were trimmed to 300 bp using FASTX-Toolkit (http://hannonlab.cshl.edu/fastx_toolkit/) and paired-end reads were merged using the merge-illumina-pairs application (https://github.com/merem/illumina-utils) with the following quality parameters: p-value = 0.03, enforce Q30 check, no ambiguous nucleotides and perfect matching to primers. An average of 227,278 merged reads per sample was obtained. Files exceeding 150,000 reads were subsampled to that amount of reads using Mothur v.1.32.0 to standardize the depth of analysis across samples, while all reads were kept for two samples in the dataset that had less than 150,000 reads (84,462 and 101,732 reads). Merged sequences between 440 and 470 bp long were kept for analysis. USEARCH v.7.0.1001 was used to remove potential chimeras and to cluster the reads into operational taxonomic units (OTUs) using a 98% similarity cut-off. Taxonomic classification at the Phylum, Family and Genus level was assigned using the Ribosomal Database Project Multiclassifier v.1.1 tool. Taxonomic classification for the OTUs was done by selecting the highest percent identity for the OTUs representative sequences (selected by the UPARSE-OTU algorithm based on read abundance) when blasted against the Greengenes database, and confirmed through NCBI Blast and RDP SeqMatch. Percent proportions were calculated based on the total number of reads per sample. Diversity metrics were calculated using MacQIIME version 1.8.0. One-way analysis of variances (ANOVA) with Tukey’s post hoc test was used to compare bacterial composition and differences in diversity between the treatments. Statistical analysis was performed using GraphPad Prism version 6.0 (GraphPad Software, La Joya, CA, USA).

## Additional Information

**How to cite this article**: Nothaft, H. *et al*. Engineering the *Campylobacter jejuni* N-glycan to create an effective chicken vaccine. *Sci. Rep*. **6**, 26511; doi: 10.1038/srep26511 (2016).

## Supplementary Material

Supplementary Information

## References

[b1] Friedman C. J., Neiman J., Wegener H. C. & Tauxe R. V. In Campylobacter (eds Nachamkim I. & Blaser M. J.) 121–38 (ASM Press, 2000).

[b2] Allos B. M. *Campylobacter jejuni Infections*: update on emerging issues and trends. Clin Infect Dis 32, 1201–6 (2001). 11283810 10.1086/319760

[b3] Mølbak K. & Havelaar A. In Campylobacter (eds Nachamkin I., Szymanski C. M. & Blaser M. J.) 151–62 (ASM Press, 2008).

[b4] Yuki N. & Odaka M. Ganglioside mimicry as a cause of Guillain-Barre syndrome. Curr Opin Neurol 18, 557–61 (2005). 16155440 10.1097/01.wco.0000174604.42272.2d

[b5] Annamalai T. . Evaluation of nanoparticle-encapsulated outer membrane proteins for the control of *Campylobacter jejuni* colonization in chickens. Poult Sci 92, 2201–11 (2013). 23873570 10.3382/ps.2012-03004

[b6] Burr D. H. . Prevention of disease in ferrets fed an inactivated whole cell *Campylobacter jejuni* vaccine. Vaccine 23, 4315–21 (2005). 16005742 10.1016/j.vaccine.2005.03.038

[b7] Huang J. L. . Intranasal immunization with chitosan/pCAGGS-flaA nanoparticles inhibits *Campylobacter jejuni* in a White Leghorn model. J Biomed Biotechnol 2010, doi: 10.1155/2010/589476 (2010). PMC2948919 20936115

[b8] Guerry P. Nonlipopolysaccharide surface antigens of Campylobacter species. J Infect Dis 176 Suppl 2, S122–4 (1997). 9396694 10.1086/513782

[b9] Zhang Q., Meitzler J. C., Huang S. & Morishita T. Sequence polymorphism, predicted secondary structures, and surface-exposed conformational epitopes of Campylobacter major outer membrane protein. Infect Immun 68, 5679–89 (2000). 10992471 10.1128/iai.68.10.5679-5689.2000 PMC101523

[b10] Pei Z. & Blaser M. J. PEB1, the major cell-binding factor of *Campylobacter jejuni*, is a homolog of the binding component in gram-negative nutrient transport systems. J Biol Chem 268, 18717–25 (1993). 8360165

[b11] Lin J., Michel L. O. & Zhang Q. CmeABC functions as a multidrug efflux system in *Campylobacter jejuni*. Antimicrob Agents Chemother 46, 2124–31 (2002). 12069964 10.1128/AAC.46.7.2124-2131.2002 PMC127319

[b12] Zeng X., Xu F. & Lin J. Molecular, antigenic, and functional characteristics of ferric enterobactin receptor CfrA in *Campylobacter jejuni*. Infect Immun 77, 5437–48 (2009). 19737895 10.1128/IAI.00666-09 PMC2786435

[b13] Logan S. M. & Trust T. J. Outer membrane characteristics of *Campylobacter jejuni*. Infect Immun 38, 898–906 (1982). 7152677 10.1128/iai.38.3.898-906.1982 PMC347834

[b14] Blaser M. J., Hopkins J. A., Berka R. M., Vasil M. L. & Wang W. L. Identification and characterization of *Campylobacter jejuni* outer membrane proteins. Infect Immun 42, 276–84 (1983). 6618667 10.1128/iai.42.1.276-284.1983 PMC264555

[b15] Hu Y., Huang J. & Jiao X. A. Screening of genes expressed *in vivo* during interaction between chicken and *Campylobacter jejuni*. J Microbiol Biotechnol 24, 217–24 (2013). 24225374 10.4014/jmb.1308.08092

[b16] Nielsen L. N. . Identification of immunogenic and virulence-associated *Campylobacter jejuni* proteins. Clin Vaccine Immunol 19, 113–9 (2012). 22155767 10.1128/CVI.05161-11 PMC3272937

[b17] Hoppe S., Bier F. F. & von Nickisch-Rosenegk M. Microarray-based method for screening of immunogenic proteins from bacteria. J Nanobiotechnology 10, 12 (2012). 22436172 10.1186/1477-3155-10-12 PMC3368735

[b18] Hu Y. . Use of *in vivo*-induced antigen technology to identify *in vivo* expressed genes of *Campylobacter jejuni* during human infection. J Microbiol Biotechnol 24, 363–70 (2013). 24346471 10.4014/jmb.1311.11019

[b19] Oakland M., Jeon B., Sahin O., Shen Z. & Zhang Q. Functional characterization of a lipoprotein-encoding operon in *Campylobacter jejuni*. PLos One 6, e20084 (2011). 21625391 10.1371/journal.pone.0020084 PMC3100323

[b20] Khoury C. A. & Meinersmann R. J. A genetic hybrid of the *Campylobacter jejuni flaA* gene with LT-B of *Escherichia coli* and assessment of the efficacy of the hybrid protein as an oral chicken vaccine. Avian Dis 39, 812–20 (1995). 8719215

[b21] Zeng X., Xu F. & Lin J. Development and Evaluation of CmeC Subunit Vaccine against *Campylobacter jejuni*. J Vaccines Vaccin 1, doi: 10.4172/2157-7560.1000112 (2010). PMC3226820 22140651

[b22] Layton S. L. . Evaluation of Salmonella-vectored Campylobacter peptide epitopes for reduction of *Campylobacter jejuni* in broiler chickens. Clin Vaccine Immunol 18, 449–54 (2011). 21177910 10.1128/CVI.00379-10 PMC3067390

[b23] Theoret J. R. . The *Campylobacter jejuni* Dps homologue is important for *in vitro* biofilm formation and cecal colonization of poultry and may serve as a protective antigen for vaccination. Clin Vaccine Immunol 19, 1426–31 (2012). 22787197 10.1128/CVI.00151-12 PMC3428391

[b24] Hacker J., Blum-Oehler G., Muhldorfer I. & Tschape H. Pathogenicity islands of virulent bacteria: structure, function and impact on microbial evolution. Mol Microbiol 23, 1089–97 (1997). 9106201 10.1046/j.1365-2958.1997.3101672.x

[b25] Huang S., Sahin O. & Zhang Q. Infection-induced antibodies against the major outer membrane protein of *Campylobacter jejuni* mainly recognize conformational epitopes. FEMS Microbiol Lett 272, 137–43 (2007). 17521366 10.1111/j.1574-6968.2007.00752.x

[b26] Pavlovskis O. R. . Significance of flagella in colonization resistance of rabbits immunized with Campylobacter spp. Infect Immun 59, 2259–64 (1991). 2050397 10.1128/iai.59.7.2259-2264.1991 PMC258004

[b27] Nuijten P. J., van der Zeijst B. A. & Newell D. G. Localization of immunogenic regions on the flagellin proteins of *Campylobacter jejuni* 81116. Infect Immun 59, 1100–5 (1991). 1705240 10.1128/iai.59.3.1100-1105.1991 PMC258373

[b28] Laniewski P. . Evaluation of the immunogenicity of *Campylobacter jejuni* CjaA protein delivered by *Salmonella enterica* sv. Typhimurium strain with regulated delayed attenuation in chickens. World J Microbiol Biotechnol 30, 281–92 (2013). 23913025 10.1007/s11274-013-1447-5 PMC3880472

[b29] Clark J. D. . Eimeria species parasites as novel vaccine delivery vectors: anti-*Campylobacter jejuni* protective immunity induced by *Eimeria tenella*-delivered CjaA. Vaccine 30, 2683–8 (2012). 22342500 10.1016/j.vaccine.2012.02.002

[b30] Wyszynska A., Raczko A., Lis M. & Jagusztyn-Krynicka E. K. Oral immunization of chickens with avirulent Salmonella vaccine strain carrying *C. jejuni* 72Dz/92 cjaA gene elicits specific humoral immune response associated with protection against challenge with wild-type Campylobacter. Vaccine 22, 1379–89 (2004). 15063560 10.1016/j.vaccine.2003.11.001

[b31] Buckley A. M. . Evaluation of live-attenuated Salmonella vaccines expressing Campylobacter antigens for control of *C. jejuni* in poultry. Vaccine 28, 1094–105 (2010). 19853682 10.1016/j.vaccine.2009.10.018

[b32] Al-Adwani S. R., Crespo R. & Shah D. H. Production and evaluation of chicken egg-yolk-derived antibodies against *Campylobacter jejuni* colonization-associated proteins. Foodborne Pathog Dis 10, 624–31 (2013). 23742296 10.1089/fpd.2012.1313

[b33] Paul N. C., Al-Adwani S., Crespo R. & Shah D. H. Evaluation of passive immunotherapeutic efficacy of hyperimmunized egg yolk powder against intestinal colonization of *Campylobacter jejuni* in chickens. Poult Sci 93, 2779–87 (2014). 25214556 10.3382/ps.2014-04234

[b34] Jones C. Vaccines based on the cell surface carbohydrates of pathogenic bacteria. An Acad Bras Cienc 77, 293–324 (2005). 15895165 10.1590/s0001-37652005000200009

[b35] Nothaft H. . Diversity in the protein *N*-glycosylation pathways among Campylobacter species. Mol Cell Proteomics 11, 1203–19 (2012). 22859570 10.1074/mcp.M112.021519 PMC3494190

[b36] Szymanski C. M. . Detection of conserved *N*-linked glycans and phase-variable lipooligosaccharides and capsules from campylobacter cells by mass spectrometry and high resolution magic angle spinning NMR spectroscopy. J Biol Chem 278, 24509–20 (2003). 12716884 10.1074/jbc.M301273200

[b37] Guerry P. . Campylobacter polysaccharide capsules: virulence and vaccines. Front Cell Infect Microbiol 2, 7 (2012). 22919599 10.3389/fcimb.2012.00007 PMC3417588

[b38] Bertolo L. . The design of a capsule polysaccharide conjugate vaccine against *Campylobacter jejuni* serotype HS15. Carbohydr Res 366, 45–9 (2013). 23261782 10.1016/j.carres.2012.11.017

[b39] Monteiro M. A. . Capsule polysaccharide conjugate vaccine against diarrheal disease caused by *Campylobacter jejuni*. Infect Immun 77, 1128–36 (2009). 19114545 10.1128/IAI.01056-08 PMC2643618

[b40] Wacker M. . N-linked glycosylation in *Campylobacter jejuni* and its functional transfer into *E. coli*. Science 298, 1790–3 (2002). 12459590 10.1126/science.298.5599.1790

[b41] Young N. M. . Structure of the *N*-linked glycan present on multiple glycoproteins in the Gram-negative bacterium, *Campylobacter jejuni*. J Biol Chem 277, 42530–9 (2002). 12186869 10.1074/jbc.M206114200

[b42] Alemka A., Nothaft H., Zheng J. & Szymanski C. M. N-Glycosylation of *Campylobacter jejuni* Surface Proteins Promotes Bacterial Fitness. Infect Immun 81, 1674–82 (2013). 23460522 10.1128/IAI.01370-12 PMC3648013

[b43] Nothaft H. & Szymanski C. M. Protein glycosylation in bacteria: sweeter than ever. Nat Rev Microbiol 8, 765–78 (2010). 20948550 10.1038/nrmicro2383

[b44] Nothaft H. & Szymanski C. M. Bacterial protein N-glycosylation: new perspectives and applications. J Biol Chem 288, 6912–20 (2013). 23329827 10.1074/jbc.R112.417857 PMC3591601

[b45] Kowarik M. . Definition of the bacterial *N*-glycosylation site consensus sequence. EMBO J 25, 1957–66 (2006). 16619027 10.1038/sj.emboj.7601087 PMC1456941

[b46] Liu D. & Reeves P. R. *Escherichia coli* K12 regains its O antigen. Microbiology 140, 49–57 (1994). 7512872 10.1099/13500872-140-1-49

[b47] Muller-Loennies S., Lindner B. & Brade H. Structural analysis of oligosaccharides from lipopolysaccharide (LPS) of *Escherichia coli* K12 strain W3100 reveals a link between inner and outer core LPS biosynthesis. J Biol Chem 278, 34090–101 (2003). 12819207 10.1074/jbc.M303985200

[b48] Reid C. W. . Affinity-capture tandem mass spectrometric characterization of polyprenyl-linked oligosaccharides: tool to study protein *N*-glycosylation pathways. Anal Chem 80, 5468–75 (2008). 18547063 10.1021/ac800079r PMC2763189

[b49] Lin J. Novel approaches for Campylobacter control in poultry. Foodborne Pathog Dis 6, 755–65 (2009). 19425824 10.1089/fpd.2008.0247 PMC3145176

[b50] de Zoete M. R., van Putten J. P. & Wagenaar J. A. Vaccination of chickens against Campylobacter. Vaccine 25, 5548–57 (2007). 17224215 10.1016/j.vaccine.2006.12.002

[b51] Szymanski C. M., Goon S., Allan B. & Guerry P. In Campylobacter: Molecular and Cellular Biology (eds Ketley J. M. & Konkel M. E.) 259–73 (Horizon Bioscience, 2005).

[b52] Szymanski C. M., Burr D. H. & Guerry P. Campylobacter protein glycosylation affects host cell interactions. Infect Immun 70, 2242–4 (2002). 11895996 10.1128/IAI.70.4.2242-2244.2002 PMC127875

[b53] Scott N. E. . Simultaneous glycan-peptide characterization using hydrophilic interaction chromatography and parallel fragmentation by CID, HCD and ETD-MS applied to the *N*-linked glycoproteome of *Campylobacter jejuni*. Mol Cell Proteomics 10, MCP201–201–MCP201–218 (2011). 10.1074/mcp.M000031-MCP201 PMC3033663 20360033

[b54] Avci F. Y., Li X., Tsuji M. & Kasper D. L. A mechanism for glycoconjugate vaccine activation of the adaptive immune system and its implications for vaccine design. Nat Med 17, 1602–9 (2011). 22101769 10.1038/nm.2535 PMC3482454

[b55] Bahrndorff S., Gill C., Lowenberger C., Skovgard H. & Hald B. The effects of temperature and innate immunity on transmission of Campylobacter jejuni (Campylobacterales: Campylobacteraceae) between life stages of Musca domestica (Diptera: Muscidae). J Med Entomol 51, 670–7 (2014). 24897861 10.1603/me13220

[b56] Sommer H. M., Heuer O. E., Sorensen A. I. & Madsen M. Analysis of factors important for the occurrence of Campylobacter in Danish broiler flocks. Prev Vet Med 111, 100–11 (2013). 23706344 10.1016/j.prevetmed.2013.04.004

[b57] Skovgard H., Kristensen K. & Hald B. Retention of Campylobacter (Campylobacterales: Campylobacteraceae) in the house fly (Diptera: Muscidae). J Med Entomol 48, 1202–9 (2011). 22238880 10.1603/me11061

[b58] Stern N. J., Bailey J. S., Blankenship L. C., Cox N. A. & McHan F. Colonization characteristics of *Campylobacter jejuni* in chick ceca. Avian Dis 32, 330–4 (1988). 3401176

[b59] Chen L., Geys H., Cawthraw S., Havelaar A. & Teunis P. Dose response for infectivity of several strains of *Campylobacter jejuni* in chickens. Risk Anal 26, 1613–21 (2006). 17184401 10.1111/j.1539-6924.2006.00850.x

[b60] Feldman M. F. . Engineering *N*-linked protein glycosylation with diverse *O*-antigen lipopolysaccharide structures in *Escherichia coli*. Proc Natl Acad Sci USA 102, 3016–21 (2005). 15703289 10.1073/pnas.0500044102 PMC549450

[b61] Linton D. . Functional analysis of the *Campylobacter jejuni N*-linked protein glycosylation pathway. Mol Microbiol 55, 1695–703 (2005). 15752194 10.1111/j.1365-2958.2005.04519.x

[b62] Alaimo C. . Two distinct but interchangeable mechanisms for flipping of lipid-linked oligosaccharides. EMBO J 25, 967–76 (2006). 16498400 10.1038/sj.emboj.7601024 PMC1409731

[b63] Heinrichs D. E., Monteiro M. A., Perry M. B. & Whitfield C. The assembly system for the lipopolysaccharide R2 core-type of *Escherichia coli* is a hybrid of those found in *Escherichia coli* K-12 and *Salmonella enterica*. Structure and function of the R2 WaaK and WaaL homologs. J Biol Chem 273, 8849–59 (1998). 9535865 10.1074/jbc.273.15.8849

[b64] Nothaft H., Liu X., Li J. & Szymanski C. M. Campylobacter jejuni free oligosaccharides: function and fate. Virulence 1, 546–50 (2010). 21178500 10.4161/viru.1.6.13801

[b65] Nothaft H., Liu X., McNally D. J., Li J. & Szymanski C. M. Study of free oligosaccharides derived from the bacterial *N*-glycosylation pathway. Proc Natl Acad Sci USA 106, 15019–24 (2009). 19706478 10.1073/pnas.0903078106 PMC2736414

[b66] Nothaft H., Liu X., McNally D. J. & Szymanski C. M. *N*-linked protein glycosylation in a bacterial system. Methods Mol Biol 600, 227–43 (2010). 19882132 10.1007/978-1-60761-454-8_16

[b67] Linton D. . Phase variation of a beta-1, 3 galactosyltransferase involved in generation of the ganglioside GM1-like lipo-oligosaccharide of *Campylobacter jejuni*. Mol Microbiol 37, 501–14 (2000). 10931344 10.1046/j.1365-2958.2000.02020.x

[b68] Guerry P. . Phase variation of *Campylobacter jejuni* 81–176 lipooligosaccharide affects ganglioside mimicry and invasiveness *in vitro*. Infect Immun 70, 787–93 (2002). 11796612 10.1128/iai.70.2.787-793.2002 PMC127662

[b69] Hendrixson D. R. A phase-variable mechanism controlling the *Campylobacter jejuni* FlgR response regulator influences commensalism. Mol Microbiol 61, 1646–59 (2006). 16899076 10.1111/j.1365-2958.2006.05336.x

[b70] Parkhill J. . The genome sequence of the food-borne pathogen *Campylobacter jejuni* reveals hypervariable sequences. Nature 403, 665–8 (2000). 10688204 10.1038/35001088

[b71] Moxon R., Bayliss C. & Hood D. Bacterial contingency loci: the role of simple sequence DNA repeats in bacterial adaptation. Annu Rev Genet 40, 307–33 (2006). 17094739 10.1146/annurev.genet.40.110405.090442

[b72] Aminian M., Sivam S., Lee C. W., Halperin S. A. & Lee S. F. Expression and purification of a trivalent pertussis toxin-diphtheria toxin-tetanus toxin fusion protein in *Escherichia coli*. Protein Expr Purif 51, 170–8 (2007). 16950635 10.1016/j.pep.2006.07.017

[b73] Masuko T. . Carbohydrate analysis by a phenol-sulfuric acid method in microplate format. Anal Biochem 339, 69–72 (2005). 15766712 10.1016/j.ab.2004.12.001

[b74] Dwivedi R., Nothaft H., Reiz B., Whittal R. M. & Szymanski C. M. Generation of free oligosaccharides from bacterial protein N-linked glycosylation systems. Biopolymers 99, 772–83 (2013). 23749285 10.1002/bip.22296

[b75] Baba T. . Construction of *Escherichia coli* K-12 in-frame, single-gene knockout mutants: the Keio collection. Mol Syst Biol 2, 2006 0008 (2006). 10.1038/msb4100050 PMC1681482 16738554

[b76] Islam K., Khalil I., Ahsan C. R., Yasmin M. & Nessa J. Analysis of immune responses against *H pylori* in rabbits. World J Gastroenterol 13, 600–6 (2007). 17278228 10.3748/wjg.v13.i4.600 PMC4065984

[b77] Wetter M. . Engineering, conjugation, and immunogenicity assessment of *Escherichia coli* O121 O antigen for its potential use as a typhoid vaccine component. Glycoconj J 30, 511–22 (2013). 23053636 10.1007/s10719-012-9451-9

[b78] Rice B. E., Lamichhane C., Joseph S. W. & Rollins D. M. Development of a rapid and specific colony-lift immunoassay for detection and enumeration of *Campylobacter jejuni*, *C. coli*, and *C. lari*. Clin Diagn Lab Immunol 3, 669–77 (1996). 8914757 10.1128/cdli.3.6.669-677.1996 PMC170429

[b79] Krumbeck J. A. . *In vivo* selection to identify bacterial strains with enhanced ecological performance in synbiotic applications. Appl Environ Microbiol 81, 2455–65 (2015). 25616794 10.1128/AEM.03903-14 PMC4357922

