# Supplementary material for: Engineering the Campylobacter jejuni N-glycan to create an effective chicken vaccine
Source: Sci Rep. 2016 May 25;6:26511. doi: 10.1038/srep26511 (PMC4879521; doi:10.1038/srep26511)
Supplement: Supplementary Information [file srep26511-s1.doc]

**Supplementary Information**

**Engineering the *Campylobacter jejuni* N-glycan to create an effective chicken vaccine**

**Harald Nothaft^1,2,6^, Brandi Davis^3^, Yee Ying Lock^3^, Maria Elisa Perez-Munoz^4^, Evgeny Vinogradov^5^, Jens Walter^1,4^, Colin Coros^3^, and Christine M. Szymanski^1,2,7^**

^1^Department of Biological Sciences, University of Alberta, Edmonton, Canada.

^2^Alberta Glycomics Centre, University of Alberta, Edmonton, Canada.

^3^Delta Genomics, Edmonton, Canada.

^4^Department of Agricultural, Food & Nutritional Science, University of Alberta, Edmonton, Canada.

^5^Human Health Therapeutics, National Research Council, Ottawa, Canada.

**Supplementary Results**

**Bioinformatic analyses of the *C. jejuni* proteome**

FASTA protein sequences from *C. jejuni* species available from the EMBL server (http://www.ebi.ac.uk/) were used to perform an amino acid motif search using the protein pattern find software Sequence Manipulation Suite: Protein Pattern Find (<http://bioinformatics.org/sms2/protein_pattern.html>), with (d|e).n.(s|t) as the search criteria that matches the requirement for the bacterial N-linked glycosylation site D/E-X1-N-X2-S/T. Since positions X1 and X2 do not tolerate a proline^1^, obtained sequences were manually investigated for the occurrence of this amino acid and excluded if present. The annotations/putative functions of the remaining proteins were subsequently (manually) searched for the keywords “periplasmic”, “membrane” and “secreted proteins” and proteins were sorted according to the frequency of glycosylation sites present.

**Cloning, expression and validation of the glycosylated GlycoTag fusion protein.**

The gene encoding an enzymatically inactive and nontoxic form of the diphtheria toxin (toxoid, *toxC*) from Corynebacterium diphtheriae was amplified from plasmid pPDT1^2^ with oligonucleotides CS-378 (5’- ATATATATCCATGGCTGCTGATGATGTTGTTGATTC-3’) and CS-379 (5’- ATATACTCGAGTCGCCTGACACGATTTCCTGCACAGG3’) to introduce NcoI and XhoI sites, respectively. The obtained NcoI-XhoI digested PCR product was inserted into plasmid pET22b cut with the same enzymes translationally fusing the gene to the plasmid-derived *pelB* secretion sequence for the transport of the product into the periplasmic space. A 271 bp DNA fragment including the 9 N-glycosylation sequon repeat (GlycoTag, GT) was amplified from chromosomal DNA of *C. jejuni* 11168 with oligonucleotides CS-334 (5’ AAACTCGAGTTCATAAAAAATTTCAAGC3’) and CS-335 (5’ ATATCTCGAGCTCTTTTTTTAATTGCG3’) inserting XhoI sites in the 5’ and 3’ prime ends. To fuse the GlycoTag sequence to the C-terminus of ToxC, the XhoI digested PCR product was inserted into plasmid pET22b*toxC,* linearized with XhoI, and dephosphorylated with shrimp alkaline phosphatase (SAP). The orientation of the GT sequence was confirmed by sequencing. This resulting construct expresses the *pelB*-*toxC*-GT fusion including a C-terminal pET22b derived Hexa-Histidine (His_6_) tag. Protein expression was performed in *E. coli* BL21(DE3) in the presence of plasmid pACYC184(*pgl*) ^3^. An overnight culture was used to inoculate 1 litre of 2xYT broth to an OD_600_ of 0.1. Cells were grown at 37°C until an OD_600_ of 0.6 was reached. Cells were cooled on ice for 30 min, protein expression was induced by addition of IPTG to a final concentration of 0.5 mM, and cells were grown for an additional 18 hrs at 30°C. Cells were cooled on ice, harvested by centrifugation (15 min 4,200 X g, 4°C), and resuspended in PBS supplemented with an EDTA free protease inhibitor cocktail according to the instructions of the manufacturer (Roche). Cells were disrupted in a cell disrupter (Constant Systems, Ltd), the resulting suspension was centrifuged for 30 min at 13,000 X g, 4°C, and the resulting supernatant was loaded onto a 1 ml Ni-NTA column using the AEKTA purification system (GE Healthcare). After an initial wash step with 10 mM imidazole in PBS, an imidazole gradient was applied from 10 mM - 250 mM over 50 column volumes. Elution fractions that contained the ToxC-GT-His_6_ protein were analyzed by 12.5% SDS-PAGE, combined and the glycosylation status of ToxC-GT-His_6_ was verified by Western blotting as described previously^4^. The ToxC-GT-His_6_ proteins were dialyzed against 25 mM potassium phosphate buffer, 10 mM NaCl, pH 7.2, and further purified by anion exchange chromatography on a 2.5 ml MonoQ column (GE Healthcare) with a 100 ml linear gradient of NaCl (10–500 mM) in 25 mM potassium phosphate, pH 7.2. Fractions containing ToxC-GT-His_6_ were desalted by size exclusion chromatography on a Sepahadex 75 column using PBS as the mobile phase. Fractions that contained the target protein as determined by 10% SDS-PAGE and Western blotting with R1-4 antisera were combined, and the concentration was determined using the Bradford assay and adjusted to 0.2 mg/ml. If necessary, centrifugal filters (Amicon, 10 kDa cut-off) were used to concentrate the proteins. Proteins were stored at 4°C until further use.

**Supplementary Methods**

***E. coli* vaccine shedding.**

The *E. coli* fecal shedding was inspected by cloacal swabs taken prior to the 1^st^ and 2^nd^ vaccine feeding and on day 2 and 7 after the first as well as on day 2 and 5 after the 2^nd^ vaccine feeding. To determine the presence of *E. coli* on the day of euthanasia, serial dilutions of the cecal contents were plated on selective LB (Kan, Cm) agar plates. After 18 hr of incubation at 37°C plates were inspected for antibiotic resistant colonies.

**Statistics.**

The one-tailed Student's t-test was used for the analysis of *C. jejuni* counts (CFU) on day 35 and for the comparison of IgY levels as detected in serum samples on day 28 in infected and non-infected chickens. P-values were calculated from comparisons of the medians obtained from each group. Statistically significant differences are indicated as follows; (*) p-value <0.05; (**) p-value <0.005 ; (ns) no significant difference, p-value >0.05.

**Supplementary Table 1. Bacterial strains and plasmids used in this study.**

| **Strain/plasmid** | **Relevant genotype, description or source** | **Reference** |
| --- | --- | --- |
| ***Plasmids:*** |  |  |
|  |  |  |
| pACYC184 | Cloning vector, Cm^R^ | 5 |
| pACYC184(*pgl_mut_*) | Encodes the *C. jejuni pgl* cluster with mutations W458A and D459A in the oligosaccharyltransferase PglB, Cm^R^ | 3 |
| pACYC184(*pgl*) | Encodes the *C. jejuni pgl* cluster, Cm^R^ | 3 |
| pET22b | IPTG-inducible, T7 promoter-dependent expression, contains *pelB* secretion signal and Hexa-Histidine-Tag, Amp^R^ | Novagen |
| pET22b-toxC-His | pET22b derivative; periplasmic expression of Hexa-Histidine-tagged ToxC from *Corynebacterium diphtheriae*, Amp^R^ | This study |
| pET22b-toxC-GT-His | pET22b derivative; periplasmic expression of Hexa-Histidine-tagged ToxC-GT, Amp^R^ | This study |
| pPDT1 | Plasmid containing full length *toxC* from *Corynebacterium diphtheriae*, Amp^R^ | 2 |
|  |  |  |
| ***Strains:*** |  |  |
| ***E. coli*** |  |  |
| K12 (BW25113) | ∆(*araD*-*araB*)567, ∆*lacZ*4787(::*rrnB*-3), lambda^-^, *rph*-1, ∆(*rhaD*-*rhaB*)568, *hsdR*514 | 6 |
| K12 (BW25113) *wzy::kan* | *E. coli* K-12 (BW25113) O-antigen polymerase mutant, Km^R^ | 7 |
| DH5α | F– Φ80*lac*ZΔM15 Δ(*lac*ZYA-*arg*F) U169 *rec*A1 *end*A1 *hsd*R17 (rK–, mK+) *pho*A *sup*E44 λ– *thi*-1 *gyr*A96 *rel*A1 | Invitrogen |
| BL21 (DE3) | F– *omp*T *hsd*SB (rB–, mB–) *gal dcm*(DE3) | Novagen |
|  |  |  |
| ***Campylobacter*** |  |  |
| *C. jejuni* | 81-176; wild-type, patient isolate used in human infection studies | 8 |
| *C. jejuni* Δ*pglB* | NCTC 11168 *pglB*::*kan,* Km^R^ | 9 |

**Supplementary Table 2.** LPS core-N-glycan NMR – chemical shifts

|  |  | 1 | 2 | 3 | 4 | 5 | 6 | 7 |
| --- | --- | --- | --- | --- | --- | --- | --- | --- |
| α-GalNAc **A** | δ_H_ | 5.47 | 4.27 | 3.23 | 4.07 | 3.92 | 3.70; 3.75 |  |
|  | δ_C_ | 98.2 | 51.0 | 68.0 | 77.6 | 72.7 | 60.8 |  |
| α-GalNAc **C** | δ_H_ | 5.13 | 4.29 | 4.19 | 4.13 | 4.49 | 3.66; 3.78 |  |
|  | δ_C_ | 98.2 | 51.5 | 67.8 | 77.4 | 71.6 | 59.9 |  |
| α-GalNAc **D** | δ_H_ | 5.07 | 4.23 | 4.04 | 4.06 | 4.39 | 3.70; 3.73 |  |
|  | δ_C_ | 99.3 | 51.4 | 68.4 | 69.6 | 71.9 | 61.8 |  |
| α-GalNAc **E** | δ_H_ | 5.04 | 4.30 | 4.15 | 4.13 | 4.43 | 3.65; 3.68 |  |
|  | δ_C_ | 99.3 | 51.5 | 67.8 | 77.4 | 72.3 | 60.5 |  |
| α-GalNAc **F** | δ_H_ | 5.02 | 4.53 | 4.17 | 4.36 | 4.45 | 3.56; 3.64 |  |
|  | δ_C_ | 99.5 | 50.5 | 67.8 | 75.6 | 72.3 | 60.3 |  |
| β-Glc **G** | δ_H_ | 4.60 | 3.32 | 3.48 | 3.38 | 3.43 | 3.71; 3.92 |  |
|  | δ_C_ |  | 74.1 | 76.8 | 70.9 | 76.9 | 61.8 |  |
| α-GlcNAc **N** | δ_H_ | 4.54 | 3.82 | 3.74 | 3.68 | 3.45 | 3.75; 3.92 |  |
|  | δ_C_ |  | 55.3 | 79.6 | 72.3 | 76.9 | 61.9 |  |
| α-Hep **L** | δ_H_ | 4.89 | 3.97 | 3.80 | 3.86 | 3.57 | 4.16 | 3.80; 4.01 |
|  | δ_C_ | 100.4 | 71.1 | 72.0 | 67.3 | 72.8 | 68.4 | 73.2 |
| α-Glc **K** | δ_H_ | 5.18 | 3.61 | 3.75 | 3.51 | 4.15 | 3.67; 3.92 |  |
|  | δ_C_ | 97.1 | 72.4 | 74.6 | 70.4 | 71.2 | 65.9 |  |
| α-Glc **I** | δ_H_ | 5.47 | 3.68 | 3.85 | 3.54 | 4.08 | 3.82; 3.90 |  |
|  | δ_C_ | 98.2 | 76.8 | 72.1 | 70.3 | 72.6 | 61.2 |  |

NMR data were recorded at 500 MHz at 25°C. Note that the anomeric carbon was not visible for G and N due to low concentrations. Chemical shifts are shown in ppm.

**Supplementary Table 3.** The *E. coli* live vaccine is self-limiting.

|  | **Swab/Day** | | | | | | |
| --- | --- | --- | --- | --- | --- | --- | --- |
| **Vaccination prior to challenge** | **7*** | **9** | **14** | **21**** | **23** | **28** | **35** |
| PBS | - | - | - | - | - | - | - |
| *E. coli* pACYC184 | - | +++ | ++ | +++ | ++ | + | - |
| *E. coli* pACYC184 (*pgl*_mut_) | - | ++ | + | - | + | - | - |

*Before 1st vaccine feeding; **before 2nd vaccine feeding

+/- indicates whether the *E. coli* vaccine is detected in cloacal swabs

**Supplementary Figure 1.**

**Supplementary Figure 1.** Confirming the presence of *C. jejuni* N-glycans after bacterial passage through a vaccinated chicken. **(A)** A representative selective agar plate with colonies obtained after plating the cecal contents from a low colonized bird after vaccination with live *E. coli* expressing the LPS core *C. jejuni*-N-glycan compound. **(B)** Reactivity with the N-glycan specific antiserum (R1-4) after transfer of the colonies (colony lift) to a PVDF membrane. Each dot represents one colony. Also, 2 μg and 10 μg of whole cell lysate of *C. jejuni* wild-type and a *C. jejuni* *pglB* mutant were spotted as positive (+ve) and negative (-ve) controls.

**Supplementary References**

1. Kowarik, M. et al. Definition of the bacterial *N*-glycosylation site consensus sequence. *EMBO J* **25**, 1957-1966 (2006).

2. Aminian, M., Sivam, S., Lee, C.W., Halperin, S.A. & Lee, S.F. Expression and purification of a trivalent pertussis toxin-diphtheria toxin-tetanus toxin fusion protein in *Escherichia coli*. *Protein Expression and Purification* **51**, 170-178 (2007).

3. Wacker, M. et al. *N*-linked glycosylation in *Campylobacter jejuni* and its functional transfer into *E. coli*. *Science* **298**, 1790-1793 (2002).

4. Nothaft, H. et al. Diversity in the protein *N*-glycosylation pathways among *Campylobacter* species. *Mol Cell Proteomics* **11**, 1203-1219 (2012).

5. Chang, A.C. & Cohen, S.N. Construction and characterization of amplifiable multicopy DNA cloning vehicles derived from the P15A cryptic miniplasmid. *Journal of Bacteriology* **134**, 1141-1156 (1978).

6. Datsenko, K.A. & Wanner, B.L. One-step inactivation of chromosomal genes in *Escherichia coli* K-12 using PCR products. *Proc Natl Acad Sci U S A* **97**, 6640-6645 (2000).

7. Baba, T. et al. Construction of *Escherichia coli* K-12 in-frame, single-gene knockout mutants: the Keio collection. *Molecular Systems Biology* **2**, 2006 0008 (2006).

8. Black, R.E., Levine, M.M., Clements, M.L., Hughes, T.P. & Blaser, M.J. Experimental *Campylobacter jejuni* infection in humans. *The Journal of Infectious Diseases* **157**, 472-479 (1988).

9. Nothaft, H., Liu, X., McNally, D.J., Li, J. & Szymanski, C.M. Study of free oligosaccharides derived from the bacterial *N*-glycosylation pathway. *Proc Natl Acad Sci U S A* **106**, 15019-15024 (2009).
